# Molecular Aspects Implicated in Dantrolene Selectivity with Respect to Ryanodine Receptor Isoforms

**DOI:** 10.3390/ijms24065409

**Published:** 2023-03-12

**Authors:** Jana Gaburjakova, Marta Gaburjakova

**Affiliations:** Institute of Molecular Physiology and Genetics, Centre of Biosciences, Slovak Academy of Sciences, Dubravska cesta 9, 840 05 Bratislava, Slovakia

**Keywords:** ryanodine receptor, dantrolene, malignant hyperthermia, skeletal muscle, cardiac muscle, failing heart, arrhythmias

## Abstract

Dantrolene is an intra-cellularly acting skeletal muscle relaxant used for the treatment of the rare genetic disorder, malignant hyperthermia (MH). In most cases, MH susceptibility is caused by dysfunction of the skeletal ryanodine receptor (RyR1) harboring one of nearly 230 single-point MH mutations. The therapeutic effect of dantrolene is the result of a direct inhibitory action on the RyR1 channel, thus suppressing aberrant Ca^2+^ release from the sarcoplasmic reticulum. Despite the almost identical dantrolene-binding sequence exits in all three mammalian RyR isoforms, dantrolene appears to be an isoform-selective inhibitor. Whereas RyR1 and RyR3 channels are competent to bind dantrolene, the RyR2 channel, predominantly expressed in the heart, is unresponsive. However, a large body of evidence suggests that the RyR2 channel becomes sensitive to dantrolene-mediated inhibition under certain pathological conditions. Although a consistent picture of the dantrolene effect emerges from in vivo studies, in vitro results are often contradictory. Hence, our goal in this perspective is to provide the best possible clues to the molecular mechanism of dantrolene’s action on RyR isoforms by identifying and discussing potential sources of conflicting results, mainly coming from cell-free experiments. Moreover, we propose that, specifically in the case of the RyR2 channel, its phosphorylation could be implicated in acquiring the channel responsiveness to dantrolene inhibition, interpreting functional findings in the structural context.

## 1. Introduction

A broad spectrum of cellular physiological processes is tightly controlled by Ca^2+^ entering the cell from the extracellular environment and/or intracellular Ca^2+^ stores such as the endo/sarcoplasmic reticulum (ER/SR). It is, therefore, only logical that Ca^2+^ signaling has been established as a hub of specific downstream physiological responses (reviewed in [1,2]). Given a critical role Ca^2+^ has, it is not surprising that Ca^2+^ is implicated in the development of many pathological processes and Ca^2+^ dysregulation has emerged as a key feature in the pathogenesis of the most often civilization diseases such as cardiovascular [3,4] and neurodegenerative diseases [5,6]. Ca^2+^ signaling is based on orchestrated interactions of Ca^2+^ influx and efflux proteins [2] and the ryanodine receptor (RyR) is one of the main components. It is an intracellular Ca^2+^ channel mediating a massive release of Ca^2+^ from the ER/SR essential for excitation–contraction coupling in striated muscle [3] as well as neurotransmitter release [7] and synaptic plasticity [8] in neurons. It has been firmly established that impaired RyR function contributes to severe cardiac and skeletal muscle myopathies, cardiac arrhythmias, and heart failure in a significant manner [9,10,11,12].

A common defect in RyR function is its hyperactivity, resulting in cytoplasmic Ca^2+^ overload [3,11,13]. Although specific inhibition of the RyR channel would have a beneficial therapeutic effect, currently, only few drugs are available to substantially reduce the RyR activity in in vivo and in vitro experiments (e.g., flecainide, Rycal S107, and dantrolene) (reviewed in [14,15]). Of these compounds with a promising therapeutic potential, dantrolene is the only clinically used agent for the effective treatment of RyR-linked channelopathy, malignant hyperthermia (MH) (reviewed in [16]). Potentially life-threatening MH has been recognized as a hereditary disorder of skeletal muscle, clinically manifested as a hypermetabolic crisis triggered by exposure to certain volatile anesthetics (e.g., halothane) [17]. Although it has been well established that all three mammalian RyR isoforms (RyR1, RyR2, and RyR3) with a specific expression pattern contain an almost identical dantrolene-binding sequence [18,19], at the functional level, dantrolene seems to act as an isoform-selective inhibitor. Whereas RyR1 and RyR3 channels are competent to bind dantrolene, the RyR2 channel is unresponsive [20,21,22,23,24,25,26,27,28,29,30]. This widely accepted view has been, however, challenged by several in vivo and in vitro studies [31,32,33,34,35], pointing to the complexity of dantrolene’s action on the RyR isoforms.

Despite dantrolene having a significantly reduced mortality from MH [36,37], this hereditary disorder remains a serious risk factor for susceptible individuals. Therefore, there is a pressing need to advance the understanding of molecular mechanisms implicated in dantrolene therapy to improve its efficiency, also considering that pharmacological approaches targeting the blockade of RyR channels by dantrolene have become a novel therapeutic approach for cardio- and neuropathologies [38]. Hence, our goal in this perspective is to provide the best possible clues to the molecular aspects of dantrolene selectivity with respect to RyR isoforms by identifying and discussing potential sources of contradictory results coming from cell-free experiments, including [^3^H]ryanodine binding, single-channel recordings, and Ca^2+^ release from isolated SR/ER microsomes derived from muscle tissues or RyR-transfected HEK-293 cells.

## 2. RyR-Linked Channelopathies

In respect of the pathogenesis of inherited conditions, more than 800 single-point mutations in the RyR channel (listed in The Human Gene Mutation Database [39]) have been associated with a number of potentially life-threating diseases. In mammalian tissues, three different RyR isoforms have been identified, sharing a sequence identity of 63–67% [40]. The RyR1 is predominantly expressed in skeletal muscle [41,42]; the RyR2 is the most abundant in cardiac muscle [43,44]; the RyR3 is expressed in a variety of tissues without a clear dominance [45,46,47]. Notably, the RyR2 isoform is the major one present in the brain [48]. Over 480 RyR1 mutations have been primarily linked to MH [49,50] and several congenital myopathies including central core disease (CCD) [51,52,53] and multi-minicore disease (MMD) [54,55]. In early studies, RyR1 mutations tended to be clustered in three distant hotspots: the N-terminal region (residues 1–600), the central region (residues 2100–2500), and the C-terminal region (residues from 3800 to the C-terminus). Currently, a number of mutations have been reported as being evenly distributed throughout the RyR1 sequence (reviewed in [49,56,57,58]). MH is a pharmacogenetic disorder in which certain volatile anesthetic agents (e.g., halothane) trigger a sudden increase in body temperature due to the abnormal metabolic and contractile activity of skeletal muscles [17]. Families susceptible to MH and having the non-myopathy phenotype harbor one of nearly 230 single-point RyR1 mutations, which are responsible for the defective RyR1 functioning, manifested by channel overactivity to volatile anesthetics [49,59,60,61,62]. Whereas MH patients do not have any phenotype without anesthesia, almost 260 RyR1 mutations are associated with various myopathies (such as CCD and MMD), which are often characterized by hypotonia and proximal muscle weakness (reviewed in [63]). As MH episodes have been reported also in CCD and MMD, all patients carrying one of the RyR1 mutations are considered potentially MH-susceptible [64].

In the RyR2 sequence, over 320 single-point mutations were identified and associated with several cardiac conditions. These mutations are mostly clustered in four hotspots: region I (roughly 80–470 residues), region II (roughly 2250–2550 residues), region III (roughly 3800–4200), and region IV (from around 4500 to the C-terminus) [58,65]. Despite such a strong clustering, the overall distribution pattern of RyR1 and RyR2 mutations appears to be similar (reviewed in [11,58,66]), suggesting similar modulatory mechanisms of RyR dysfunctions. The vast majority of almost 170 RyR2 mutations encode for catecholaminergic polymorphic ventricular tachycardia, type 1 (CPVT1) [67,68] and the remaining mutations are mainly associated with arrhythmogenic right ventricular dysplasia, type 2 (ARVC/D2) [69,70] and polymorphic ventricular tachycardia (PVT) [71,72]. CPVT1 is a malignant arrhythmogenic disorder that is observed in individuals with a structurally normal heart who present ventricular arrhythmias when exposed to stress [67,69,73]. It is widely accepted that mutations in the RyR2 channel lead to a diastolic Ca^2+^ leakage from the SR, particularly under stress, causing vulnerability to ventricular arrhythmias (reviewed in [11,73,74,75]). ARVC/D2 is a stress-induced arrhythmogenic disorder characterized by right ventricular cardiomyopathy [76,77]. Again, an imbalance in Ca^2+^ homeostasis as a result of RyR2 mutations has been suggested to be the main molecular mechanism involved in electrical instability [69], also contributing to progressive replacement of cardiomyocytes by fat and fibrous tissue [78,79].

The RyR3 channel is the least studied isoform, and consequently little is known of its potential dysfunction caused by single-point mutations. A few recent studies have linked RyR3 mutations with gender dysphoria [80] or Alzheimer’s disease [81], but the molecular mechanisms implicated have not yet been explored.

## 3. The Dantrolene-Mediated Inhibitory Effect on the RyR1 Channel

### 3.1. Sensitivity of the RyR1 Channel to the Activation Effect of Volatile Anesthetics

It is well known that, in MH-susceptible individuals, Ca^2+^ release from the skeletal SR via mutated RyR1 channels is hypersensitive to certain volatile anesthetics [49,59,60,62], and to caffeine as well [82,83,84]. While still far from complete, we are slowly beginning to understand molecular mechanisms underlying this condition. The general unifying picture of MH derived from cell-free experiments is that MH mutations make the RyR1 channel hypersensitive to halothane concentrations ranging from several tens to several hundreds of µM [85,86]. No specific conditions were required to induce such activation, in contrast to the wild-type RyR1 channel [85,87,88,89]. The clinically used concentration of halothane is unlikely to exceed ~1 mM [90,91,92]. Thus, the MH RyR1 activation monitored in cell-free experiments occurred in the therapeutic range. In the case, when conditions mimicked the cell situation mainly in respect to ATP, it is evident that even the wild-type RyR1 channel became substantially responsive to clinically relevant halothane concentrations [87,88,89]. However, an MH episode was not a clinically observed phenomenon in healthy individuals. Duke et al. [93,94,95] suggested that it could be a consequence of a potent inhibitory action of a physiological concentration of cytosolic Mg^2+^ (~1 mM) [96]. Consistent with this hypothesis, RyR1 activation by halothane was indeed antagonized by 1 mM cytosolic Mg^2+^ [89,97]. This might have profound clinical consequences. Conditions resulting in hypomagnesaemia (the cytosolic Mg^2+^ concentration drops below 0.5 mM [98]) would inevitably increase the occurrence of an MH event even in healthy individuals [94,95,99]. Thus, maintenance of a normal level of cytosolic Mg^2+^ seems to be fundamental for stabilizing the wild-type RyR1 channel affected by halothane in a closed state. In MH, the Mg^2+^ situation is more complex because both reduced Mg^2+^ sensitivity of the RyR1 channel [100,101] caused by a MH mutation and/or a decreased level of cytosolic Mg^2+^ [102] might come into play. Considering current experimental evidence, it is fair to suggest that the confluence of both factors might contribute to MH susceptibility.

### 3.2. Structural Determinants of Halothane Binding to the RyR1 Channel

Despite the use of volatile anesthetics being widespread in surgical practice, a detailed understanding of the molecular mechanism and site of their action is still lacking. Based on the current experimental and computational evidence provided for the K^+^ channels [103,104] and nicotinic acetylcholine receptors [105,106], it can be speculated that the halothane–RyR1 interaction may be controlled primarily by hydrophobic forces and halothane may bind to a hydrophobic cavity, presumably situated near the RyR1 membrane domain. The enhanced responsiveness of the MH RyR1 channel to halothane appears to be a direct consequence of conformational changes exerted by an MH mutation. Earlier, the domain switch model has been proposed to explain RyR1 activation in normal skeletal muscle as well as RyR1 dysfunction in MH [107,108,109]. In this model, the N-terminal domain and Helical domain1 as two major RyR1 regions clustering many of the MH mutations interact with each other to form a domain switch that is critical in stabilizing the channel closed state. MH mutations in either the N-terminal domain or Helical domain1 weaken the tight inter-domain interactions, causing partial “unzipping” of the proposed switch. As a result, the closed state of the channel is destabilized, leading to RyR1 hypersensitivity to activation by various stimuli. Others have indeed reported that various MH mutations stimulate RyR1 sensitivity to cytosolic Ca^2+^ [82,110,111,112,113], luminal Ca^2+^ [61,82,87], ATP [100], and/or caffeine [100,114], using various cell-free approaches. Recent studies have provided cryo-EM characterization of two MH RyR1 mutants (R164C, rabbit numbering; R615C, porcine numbering), which do not support the interdomain hypothesis [112,113]. Although the N-terminal domain has multiple interactions with the Helical domain1, they are preserved despite the presence of MH mutations. Overall, the MH RyR1 mutants adopt a conformation between fully open and closed, thus facilitating the RyR1 over-reactivity. Because defective cytosolic Mg^2+^ handling could be expected to influence the probability of an MH episode, we ask how a macroscopic manifestation could be reflected at the structural level. 

### 3.3. Essential Conditions for Observing the Dantrolene-Mediated Inhibitory Effect on the RyR1 Channel

As mentioned earlier, dantrolene is a key component in the treatment of an MH crisis, which occurs by antagonizing the activating effect of volatile anesthetics on the RyR1 channel. After dantrolene was introduced, the mortality of MH decreased from 80% down to 6−10% [36,37], demonstrating the high efficacy of dantrolene in MH-susceptible patients. Such a strong action of dantrolene has been consistently demonstrated also in vitro, particularly at the cellular level when contractility and/or Ca^2+^ signaling were investigated in a whole skeletal muscle fascicle or single skeletal muscle cells [28,115,116,117,118]. However, in cell-free experiments, a lot of controversy surrounds the dantrolene-mediated inhibition of the RyR1 channel. Apparently, over 50 years of studies on dantrolene is not enough to give a clear and consistent picture of this issue.

In a large body of literature, four main factors have appeared to condition dantrolene’s action. Specifically, the presence of ATP, cytosolic Mg^2+^, and calmodulin (CaM) together with the increased temperature (35−37 °C) were required (either alone or in various combinations) for the dantrolene effect on the RyR1 channel (Table 1). All of them are physiologically relevant. Unexpectedly, experimental data are so heterogenous that it is still a challenge to identify what factors are unnecessary or insufficient. In an attempt to approach this goal, we carefully evaluated the role of some additional aspects and variables, not yet considered, which might have a substantial impact on dantrolene’s action. Although many groups added dantrolene solely to the wild-type [28,89,118,119,120,121,122] or MH RyR1 channels [87,97], it was certainly not a source of such variability, because Fruen et al. [29] and Zhao et al. [30] clearly demonstrated the inhibition of both wild-type and MH RyR1 channels, testing the same experimental conditions. Furthermore, the absence and presence of dantrolene-mediated inhibition were reported in each of the numerous cell-free studies [28,29,30,119,121,122], when experimental conditions were changed only in respect to four aforementioned factors, indicating that failure of dantrolene was not related to its time-dependent instability in aqueous solutions [123]. Moreover, the complete RyR1 irresponsiveness to dantrolene reported in [89,97,118,120] was not caused by the low concentrations tested (10−40 µM), because dantrolene in the similar concentration range (10−50 µM) has been shown to substantially reduce RyR1 activity [28,29,30,87,119,121,122]. In addition, the values of the dissociation constant (K_d_) are in the nanomolar range (Table 2) [18,124,125], pointing to the high-affinity dantrolene–RyR1 interaction. Notably, the clinically relevant concentration of dantrolene is approximately 10 µM [126].

The other reason for data heterogeneity may stem from different concentrations of ATP, cytosolic Mg^2+^, and calmodulin considering the strong sensitivity of the RyR1 channel to ATP activation [127,128,129], Mg^2+^-dependent inhibition [130,131,132], and CaM-dependent regulation [133,134]. This implies that these three factors might potentiate the effect of dantrolene as they directly interact with the RyR1 channel. When cytosolic Mg^2+^ was added, its concentration varied from 0.25 mM to 3 mM. Although dantrolene only failed to inhibit the RyR1 channel at low Mg^2+^ (0.25 mM [89] and 0.45 mM [29]), cytosolic Mg^2+^ appears to be unnecessary in several other studies [30,119,121]. With respect to ATP concentration, the situation is similar because ATP was not a prerequisite for RyR1 inhibition [121], and furthermore, when ATP was added, the occurrence of dantrolene-mediated inhibition was not dependent on ATP concentration, ranging from 100 µM to 5 mM. CaM, a ubiquitous Ca^2+^-binding protein, has also been found to condition dantrolene’s action. It confers Ca^2+^-dependent regulation on many proteins (reviewed in [135]), and particularly in the case of the RyR1 channel, it shifts the channel sensitivity to cytosolic Ca^2+^ to lower concentrations [133,134]. From all studies listed in Table 1, only Diszházi et al. [122] tested the presence of endogenous CaM in their skeletal SR samples by Western blotting. They clearly showed that endogenous CaM was completely detached from the RyR1 channel complex, obviously during an isolation procedure. Because the CaM-RyR1 interaction appears to be so delicate, it is feasible to assume that endogenous CaM was always missing, and to restore the CaM-RyR1 interaction, exogenous CaM had to be added to skeletal SR samples. Making such an assumption, we can conclude that CaM is not a critical requirement for dantrolene’s action, because RyR1 inhibition was also observed in the absence of CaM [28,87,119,122]. In addition, 100 nM [121,122] as well as 1−2 µM exogenous CaM [29,30,118,119] failed to induce RyR1 responsiveness to dantrolene in each experiment. Similarly, a rise in temperature by ~10 °C over a room temperature was not found to be a causal factor conditioning a consistent dantrolene-mediated inhibition. In summary, it seems reasonable to conclude that ATP, cytosolic Mg^2+^, CaM, and the increased temperature only together are capable of fully driving dantrolene’s action, as RyR1 inhibition was a consistent observation when all of such physiological factors were involved together [29,30]. On the other hand, failure of dantrolene was always observed when neither of them was present [28,89,97,121].

## 4. The RyR2 Channel as an Endogenous Target of Dantrolene

### 4.1. Activation of the RyR2 Channel by Volatile Anesthetics

The depression of cardiac contractility during surgery, when volatile anesthetics are often used, is a well-established phenomenon with a potential beneficial impact [136,137,138]. More specifically, the contractility of isolated cardiac tissue [139,140,141] and isolated cardiomyocytes [142,143,144] was directly affected, suggesting that volatile anesthetics interact with the main components of cardiac Ca^2+^ signaling. Indeed, there are several reports showing reduced SR Ca^2+^ load [142,145,146,147], presumably as a result of decreased Ca^2+^ uptake to the SR [143] and/or enhanced Ca^2+^ release from the SR via the RyR2 channel [86,89,92,148]. Cell-free experiments provided clear evidence that the RyR2 channel can be activated by various volatile anesthetics at clinically relevant concentrations (~1 mM) [89,92,148]. From them, halothane appears to be the most potent activator, while activation resulting from desflurane and enflurane was less impressive. In addition, isoflurane and sevoflurane almost completely failed to induce RyR2 activation [86,92,148]. Compared to the RyR1 channel, it is evident that the RyR2 channel is much less sensitive to volatile anesthetics. On the other hand, ATP and cytosolic Ca^2+^ (subactivating concentrations) were not required for halothane to be a potent RyR2 activator [89,92], as was otherwise seen for the RyR1 channel [87,88,89,97,149]. Both RyR isoforms, however, share a strong dependence of halothane’s action on luminal Ca^2+^concentration [89,92]. As RyR2 activation by halothane was strongly inhibited by a physiological concentration of cytosolic Mg^2+^ [92], a situation to occur in diastole, it is unlikely to play a significant role in commonly observed anesthetic-induced cardiac arrhythmias [150]. Moreover, RyR2 activation by volatile anesthetics is unlikely to contribute to cardiac depression during surgery because isoflurane and sevoflurane have similar depressant effects as halothane, albeit unlike halothane, they did not activate the channel in cell-free studies [92]. Evidently, other Ca^2+^ transporting components of Ca^2+^ signaling situated in the plasma membrane such as the L-type Ca^2+^channel [151,152,153], Na^+^/Ca^2+^exchanger, and store-operated Ca^2+^ entry [154,155] appear more likely to mediate the depressant action of volatile anesthetics. 

### 4.2. RyR2 Responsiveness to Dantrolene in the Normal Heart

It has been known for a long time that the intravenous administration of dantrolene does not seriously affect cardiovascular function [20,21,22,24,156]. Therefore, dantrolene became a favorable choice for the safe treatment of MH-susceptible patients, as it acts only on skeletal muscle. Later, numerous in vitro studies strongly supported this finding by showing little or no significant effect of dantrolene on the contractility of excised cardiac tissues, the electrical activity of isolated cardiomyocytes, or Ca^2+^ signaling in HEK293 cells expressing the wild-type RyR2 channel [23,24,157,158,159]. The strongest evidence for RyR2 resistance to dantrolene was provided by cell-free experiments [29,30]. As mentioned for the RyR1 channel, various experimental conditions were also tested for the RyR2 channel (Table 3), and unsurprisingly, a lack of the dantrolene-mediated inhibition (10 µM concentration was tested) was observed, even when ATP analog (2−3 mM), cytosolic Mg^2+^ (0.35 mM), CaM (1 µM), and the increased temperature were involved together [29]. This contrasts sharply with the RyR1 channel, whose inhibition by dantrolene was clearly expressed under such multifactorial conditions. It is worth noting that the view of RyR2’s unresponsiveness to dantrolene has been contradicted by two recent papers by the same group [92,121]. These results of cell-free experiments are, however, in stark contrast to clinical and cell-based observations [20,21,22,23,24,156,157,158,159]. Taken together, the molecular reason for broadly evidenced dantrolene selectivity is still poorly understood because the dantrolene binding site has been situated in both RyR1 and RyR2 sequences [19,160], although the binding efficacy for the RyR2 channel is almost negligible in the nanomolar range (Table 2) [19,125]. Structural aspects of this issue, also with respect to the controversial piece of data in [92,121], are discussed in Section 6. 

### 4.3. RyR2 Responsiveness to Dantrolene under Pathological Conditions

Despite RyR2’s insensitivity to dantrolene, the presence of the dantrolene-binding sequence in the RyR2 region [160] suggests that the native channel conformation restricts the access of dantrolene to its binding site. What could be the molecular nature of this barrier? Is it possible to bypass it and make the RyR2 channel capable of interacting with dantrolene? Paul-Pletzer et al. [19,160] hypothesized that associated proteins could constitute a physical obstacle or post-translational modifications could negatively affect the availability of the binding site. It has been known for a while that dantrolene improved contractile function in patients with heart failure [161] and animal models of cardiomyopathy [31,162,163,164,165]. Accordingly, dantrolene reduced arrhythmias in patients [166] and different arrhythmia models [32,33,34,167,168,169,170]. Plenty of studies demonstrated that multisite RyR2 hyperphosphorylation plays a significant role in the pathogenesis of cardiac disease; however, it has long been a highly controversial area. Even cell-free experiments demonstrated several functional modifications including increased RyR2 sensitivity to cytosolic and/or luminal Ca^2+^ or decreased sensitivity to cytosolic Mg^2+^ (reviewed in [171,172,173]). The RyR2 channel harbors three major phosphorylation Serines (S), namely S2808, S2814, and S2031 (human numbering). S2808 and S2814 are situated close to each other in the phosphorylation loop within the larger phosphorylation domain [174] and S2031 is placed at a distance of 10 nm in 3D space. While in vivo S2808 and S2031 are substrates for cAMP-dependent protein kinase (PKA), S2814 is phosphorylated by Ca^2+^/calmodulin-dependent kinase II (CaMKII) (reviewed in [173]). Although both kinases are mediators of β-adrenergic signaling in the heart, PKA appears to be a prominent one (reviewed in [175,176,177]). The working hypothesis of a tight connection between RyR2 phosphorylation and dantrolene’s action was already supported by an early work of Meyler et al. [178], showing dantrolene-dependent reduction of cardiac contractility when adrenalin was used to stimulate β-adrenergic signaling. Addressing this issue in a more specific way, Sufu-Shimizu et al. [179] demonstrated a significant attenuation of aberrant Ca^2+^ release in isolated cardiomyocytes following dantrolene treatment in CaMKIIδc-overexpressing mice. Because elevated phosphorylation at RyR2 S2814 as a consequence of chronic CaMKIIδc activation was not affected by dantrolene application, it is highly likely that the phosphorylation event at S2814 induced such conformational changes, which strongly influenced the ability of dantrolene to bind. Notably, the phosphorylation status of RyR2 S2808 seems to not play a role, because it was not changed in CaMKIIδc-transgenic mice. This conclusion was, however, contradicted by Si et al. [180], who clearly evidenced that RyR2 hyperphosphorylation at S2808, but not at S2814, was essential for the therapeutic effect of azumolene (an active analog of dantrolene) on ischemia- and reperfusion-induced arrhythmias in the rabbit heart model. Considering our recently proposed concept of “qualitative substitutability”, when alterations in RyR2 function caused by phosphorylation at S2808 or S2814 can qualitatively overlap [173], it is feasible to assume that structural consequences of such individual phosphorylation events, occurring close to each other, are very similar. This argumentation offers a simple reconciliation of conflicting results reported by Si et al. [180] and Sufu-Shimizu et al. [179], even though the concept of “qualitative substitutability” demands further experimental testing at structural as well as functional levels. To find direct evidence of RyR2 inhibition by dantrolene in failing hearts, we searched all available literature but found only the study of Kobayashi et al. [31] employing a cell-free approach. Expectedly, dantrolene (1−10 µM) abolished the spontaneous activity of the RyR2 channel when isolated from failing hearts. Moreover, they provided direct evidence for a critical role of RyR2 phosphorylation in the channel sensitization to dantrolene, activating endogenous PKA attached to the channel by cAMP.

As aforementioned, dantrolene also has a strong antiarrhythmic effect. Several studies have shown that dantrolene inhibited Ca^2+^ release from the SR isolated from hearts of transgenic mice with a CPVT1 mutation [32,167] or induced pluripotent stem cells, which were generated from CPVT patients [33,166]. Cell-free experiments have indicated that a vast majority of tested CVPT1 mutations activate the RyR2 channel in response to elevated luminal Ca^2+^ [181,182,183,184]. Although hypersensitivity to cytosolic Ca^2+^ and hyposensitivity to cytosolic Mg^2+^ could also participate [112,185,186,187,188], more frequent spontaneous Ca^2+^ release under conditions of store Ca^2+^ overload (SOICR) represents a common defect caused by CPVT1 mutations (comprehensively reviewed in [75,189,190]. This inappropriate RyR2 activation results in diastolic SR Ca^2+^ leak, which has been suggested to trigger fatal cardiac arrhythmias [191]. From a structural point of view, a widely held hypothesis states that weakened N terminal-Helical domain1 interactions are a common point in RyR2 dysfunction [32,192]. Although this zipping/unzipping concept seems to be reasonable, more cryo-EM 3D structures of the RyR2 channel with CPVT1 mutations are needed to obtain a more comprehensive view of pathological mechanisms involved [66,193]. Presently, only two works reported conformational changes caused by the CPVT1 mutations R2474S (human numbering) [194] and R176Q (human numbering) [112]. However, N terminal-Helical domain1 interactions were not a significant focus in these studies. As dantrolene abolished arrythmias in CPVT1, the possibility also exists that CPVT1 mutations might be critical for the inhibitory effect of dantrolene on the RyR2 channel, presumably by inducing favorable structural changes. This possibility is obviously not true for MH mutations, because even the wild-type RyR1 channel is substantially inhibited by dantrolene [28,29,30,119,121,122]. RyR1 and RyR2 isoforms share a sequence identity of 63%–67% [40]; thus, it seems reasonable to assume that the effects of mutations could be transduced in an isoform-specific manner [112]. The antiarrhythmic action of dantrolene in CPVT1 has been, however, documented only under stress conditions [32,33,166,167], when RyR2 channels became phosphorylated [195,196]. To dissect the role of CPVT1 mutations, cell-free experiments are necessary to test whether the mutated RyR2 channel is competent to bind dantrolene even under resting conditions.

## 5. The Inhibitory Effect of Dantrolene on the RyR3 Channel

The dantrolene-binding sequence, nearly identical to that found in the RyR1 channel, has also been identified in the RyR3 isoform. The in vivo sensitivity to dantrolene has not yet been clarified, because the RyR3 channel is ubiquitously expressed in many cells without a clear dominance [45,46,47]. There is, however, one study that addressed this issue in HEK293 cells solely overexpressing the RyR3 isoform. Using a cell-free approach, Zhao et al. [30] demonstrated a substantial RyR3 inhibition as a consequence of RyR3’s capability to interact with dantrolene. Although the value of K_d_ is still missing, it is reasonable to assume that the RyR3 channel possesses a comparable dantrolene-binding affinity to that found for the RyR1 channel (Table 2) because both isoforms showed a similar extent of inhibition by 10 µM dantrolene [30]. For the RyR3 channel, this inhibitory action was also dependent on the presence of ATP analog (2 mM) and CaM (1 µM) together with the increased temperature to 37 °C (Table 4). Of note, cytosolic Mg^2+^ was not required, as also shown for the RyR1 channel in some studies [30,119]. Under identical conditions, which were sufficient to drive RyR3 inhibition by dantrolene, the heterologously expressed RyR2 channel was not affected. This particular result further strengthens the experimental evidence of dantrolene selectivity with respect to the RyR isoforms.

## 6. Localization of the Binding Site for Dantrolene in the Three-Dimensional (3D) Structure of RyR Isoforms

**Figure 1:**
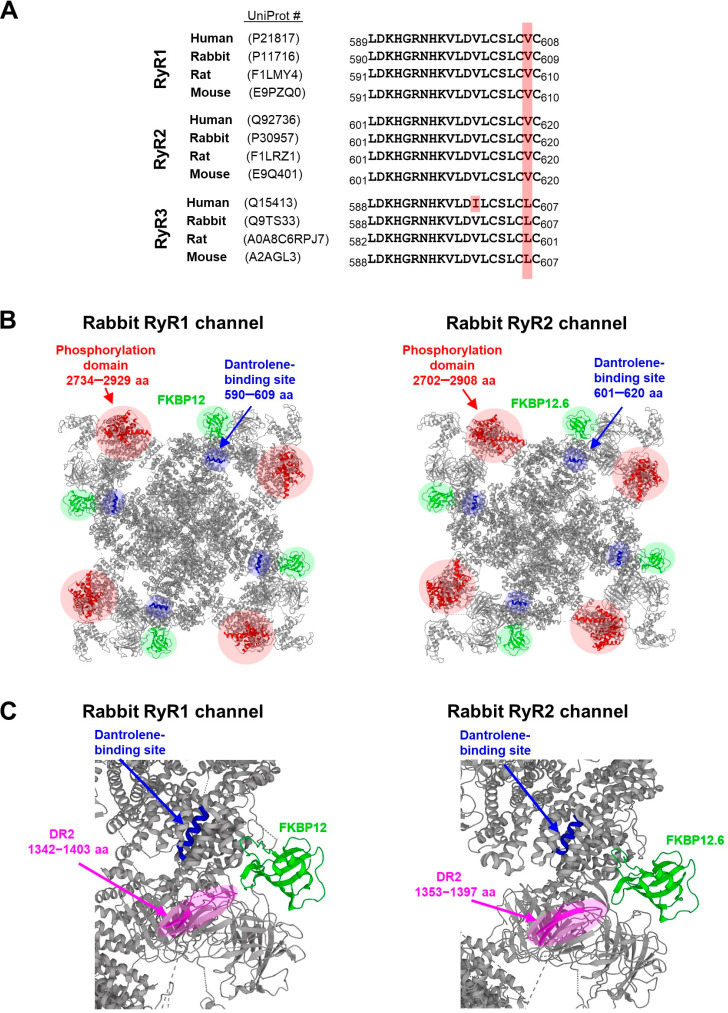
Structural aspects of dantrolene interaction with RyR isoforms. (**A**) A multiple-sequence alignment of the dantrolene-binding sequences located within all three RyR isoforms [19,160] from indicated mammals. The non-conserved residues are boxed in red. The sequences were taken from the UniProtKB database. (**B**) Cryo-EM 3D maps (cytosolic view) of the rabbit RyR1 (left) and the rabbit RyR2 (right) channels. The dantrolene-binding site is shown in blue [19,160], the phosphorylation domain in red [174], and FKBP12/12.6 in green [197,198]. (**C**) Zoomed-in view of the DR2 in one of four RyR1 (left) and RyR2 (right) subunits shown in magenta [199]. As the DR2 region is structurally unresolved, we highlighted two β-sheets within RyR1 (β1:1287−1290 aa and β2:1438−1439 aa), as well as RyR2 (β1:1300−1304 aa and β2:1426−1431 aa) subunits that closely surround the DR2 region. The dantrolene-binding site in blue and FKBP12/12.6 in green are also shown. The cryo-EM 3D structure of RyR1 (PDB ID: 3J8H [198]) and RyR2 channels (PDB ID: 5L1D [197] were taken from the Protein Data Bank available at rcsb.org [200]. UniProt #, UniProt accession number.

To uncover the molecular components implicated in the selectivity of dantrolene’ action, structural findings should be considered because they offered valuable insights in many cases. An early effort at mapping the dantrolene-binding site on the RyR1 channel, [^3^H]azidodantrolene, a photoaffinity analog of dantrolene, has been utilized to identify the RyR1 region responsible for interaction [160]. The RyR1 sequence comprising residues 590−609 (rabbit numbering) was only specifically labeled by [^3^H]azidodantrolene when several synthetic peptides derived from the N-terminal domain of the RyR1 channel were tested. This finding was further validated on the full-length RyR1 protein heterologously expressed in CHO cells. The RyR1 sequence capable of interacting with dantrolene is fully conserved across selected mammals (Figure 1A), often used as a source of skeletal muscle tissue for cell-based as well as cell-free experiments, implying similar binding properties. A multiple-sequence alignment of all three RyR isoforms revealed the occurrence of an identical sequence in the equivalent RyR2 region encompassing positions 601−620 (rabbit numbering) (Figure 1A). Again, this sequence is fully conserved across selected mammals. Although the full-length RyR2 protein (not affected by post-translational modifications) is not competent to bind dantrolene, a shorter synthetic peptide covering the RyR2 dantrolene-binding sequence was photolabeled with [^3^H]azidodantrolene [19]. This observation provided a strong rationale for the hypothesis of a poor accessibility of the dantrolene-binding site in the native full-length RyR2 protein [19]. The RyR3 isoform differs from the other two isoforms by a single amino acid substitution (Valine for Leucine) at position 606 (Figure 1A). Sequence variability does not, however, necessarily indicate 3D structural variability and, thus, a changed binding ability. This expectation correlates extremely well with functional data showing a great RyR3 sensitivity to dantrolene inhibition, at least for the rabbit RyR3 channel [30]. We found one additional replacement of Valine with Isoleucine in the human sequence at position 600. It will be of great interest to identify its potential effect on dantrolene’s binding affinity and efficacy.

To gain further insight into the structural basis of dantrolene’s action, Wang et al. [201] attempted to map GFP-labeled Arginine 626, close to the dantrolene-binding sequence, and Tyrosine 846, downstream of the dantrolene-binding sequence, in the cryo-EM 3D structure of the murine RyR2 channel. In combination with FRET analysis, they provided several lines of evidence converging to the conclusion that the dantrolene-binding sequence is located near the FKBP12.6-binding site. FKBP12.6 (also known as calstabin 2) is a peptidyl-prolyl cis-trans isomerase stabilizing the RyR2 channel in a closed state, particularly important in diastole, and thus preventing the aberrant diastolic Ca^2+^ leak from the SR [191,202,203,204], otherwise seen in failing hearts (reviewed in [205,206,207]). Similarly, the RyR1 channel interacts with FKBP12 (also known as calstabin 1) [208,209], a phenomenon required to stabilize open/closed states of the channel [210,211]. Although the accurate identification of the RyR1 and RyR2 binding sequences for FKBP12 and FKBP12.6, respectively, is still lacking, progress was made in determining the cryo-EM 3D structure of both isoforms complexed with their corresponding FKBP12 protein [197,212]. FKBP12 or FKBP12.6 was placed on the surface area formed by the Handle, SPRY1, and SPRY3 domains (Figure 1B). To explore the spatial relationship between the dantrolene- and FKBP12/12.6-binding regions, we visualized the dantrolene-binding sequence in the cryo-EM 3D structure of the rabbit RyR1 and RyR2 channels. Despite the lack of direct structural evidence supporting this interaction site, it is clearly visible that this sequence is located at the top periphery of the cytosolic domain, in close vicinity to the FKBP12/12.6-binding site. Thus, it is not unreasonable to propose that FKBP12.6 is somehow implicated, presumably via short-range allosteric interactions, in conferring RyR2 resistance to dantrolene (Figure 1B). The abundance of existing functional data accumulated to date, which pointed to a role of RyR2 phosphorylation in acquiring RyR2 sensitivity to dantrolene, supports this hypothesis. To explain, several studies reported that phosphorylation at S2808 (located within the phosphorylation domain [174]) dissociated FKBP12.6 from the RyR2 channel [195,213,214,215]. Although this topic has been a controversial issue for a long time, searching for molecular components of dantrolene selectivity might also provide a novel mechanistic insight into a crosstalk between RyR2 phosphorylation and FKBP12.6 binding, not yet widely accepted. Thus far, no significant physical interaction between domains, where these two processes take place, was detected by Yuchi et al. [174]; however, these influential authors speculated that an allosteric control of FKBP12.6 binding might occur. A similar allosteric coupling might exist between the RyR2 phosphorylation domain and dantrolene-binding site, as dantrolene binds in close proximity to the FKBP12.6-binding site (Figure 1B). Taken together, a broad spectrum of functional data interpreted in the context of cryo-EM 3D structures suggests that the dantrolene-binding site on the RyR2 channel is conformationally sensitive. Coming back to conflicting cell-free studies reporting dantrolene-mediated inhibition of the RyR2 channel isolated from healthy hearts [92,121], the most likely explanation in the light of the above conclusion is that RyR2 channels in those studies were subjected to a certain modification, presumably during heart excision and/or channel isolation, which resulted in such conformational changes favoring dantrolene binding. This possibility should be kept in mind rather than ignored.

In the case of the RyR1 channel, its phosphorylation appears not to be a prerequisite for dantrolene’s action. There is evidence that the recombinant RyR1 channel expressed in CHO cells, evidently not phosphorylated, was competent to bind dantrolene [29]. This functional difference between RyR1 and RyR2 channels does not correlate with the structural differences, because the only known phosphorylatable site on the RyR1 channel, S2843 (rabbit numbering) [216], is located within the phosphorylation domain that is equivalent to that found in the RyR2 channel [174] (Figure 1B). As mentioned earlier, the sequence identity between all three RyR isoforms is 63–67% [40]. The largest differences are clustered in three “divergent regions”: DR1 (4254–4631 residues), DR2 (1342–1403 residues), and DR3 (1872–1923 residues) (RyR1 numbering). When we visualized them in the cryo-EM 3D structure of the rabbit RyR1 and RyR2 channels, we found that the DR2 region is located close to the dantrolene-binding site (Figure 1C). For now, we can only speculate that the DR2 region may be implicated in conferring RyR2 resistance to dantrolene and its structural impact may be antagonized by RyR2 phosphorylation. Notably, cell-free studies listed in Table 1 clearly demonstrate that the dantrolene–RyR1 interaction also requires particular RyR1 conformations as the presence of ATP, cytosolic Mg^2+^, and calmodulin together with the increased temperature (35−37 °C) was found to fully drive the channel’s responsiveness to dantrolene. The binding locations of ATP and calmodulin were mapped to the close-transmembrane region and the lateral face of the cytosolic domain, respectively, in the cryo-EM 3D structures (reviewed in [66]). Apparently, these regions are located far away from the dantrolene-binding site, strongly suggesting that a long-range allosteric coupling is involved in a potentiation of dantrolene’s action. Because the RyR1 and RyR2 channels share considerable sequence and structural homology, it is reasonable to expect that similar allosteric propagation pathways will exist in the phosphorylated RyR2 channel.

## 7. Dantrolene Suitability for Novel Clinical Applications

Dantrolene is now widely studied as a novel treatment for cardio- and neuropathologies, targeting the inhibition of RyR channels. However, the poor water solubility of dantrolene might preclude its use for effective therapy because this property often leads to inadequate and variable bioavailability. However, dantrolene still remains the most effective therapeutic agent for MH. In an emergency situation, a dantrolene solution has to be warmed to improve water solubility [217]. However, any delay in the intravenous administration of dantrolene was associated with increased morbidity and mortality [218,219]. A potential improvement has become available in the form of azumolene, a 30-fold more water-soluble analog [220]. Azumolene has been demonstrated to be as effective as dantrolene in reversing an MH crisis in MH-susceptible pigs [221,222]. Although azumolene seems to be more suitable for clinical use, its shelf life is only 2 years when in powder and 1 month when dissolved in water. To overcome this disadvantage, the dantrolene nanosuspension with improved water solubility and an approved 3-year shelf life was developed and approved for clinical practice in the year 2014 [223]. The important benefits of this nanosuspension such as short preparation time and a more rapid rate of the intravenous administration should compensate for a higher cost. Translation of the dantrolene nanosuspension to clinical application in respect of the treatment of cardiac- and neuropathologies would face many challenges. For example, its suitability for oral administration would be of particular importance. Moreover, although inhibition of the RyR2 channel in the heart could be a promising anti-heart failure or anti-CPVT1 approach, it could give rise to serious extra-cardiac side-effects because of a strong inhibitory effect of dantrolene on RyR1 and RyR3 channels. Thus, a successful drug design, appropriate formulation, and drug delivery system need to be carefully considered. In the case of neuropathologies, the situation is more favorable because intranasal administration of dantrolene could provide selective penetration into the brain without interfering with other organs expressing at least one RyR isoform and concurrently by passing the blood–brain barrier [224,225].

## 8. Conclusions and Future Outlook

Dantrolene is the only approved inhibitor of the RyR channel, currently used to truncate acute MH episodes. Although all three mammalian RyR isoforms share considerable sequence and structural homology, dantrolene was confirmed to bind only to RyR1 and RyR3 isoforms. The RyR2 isoform appears to be unresponsive, despite having the functional dantrolene-binding sequence. Numerous in vivo and in vitro studies, however, indicate that under certain pathophysiological conditions, the RyR2 channel acquires a sensitivity to dantrolene-mediated inhibition. In our view, RyR2 phosphorylation accompanied with the release of FKBP12.6 from a channel complex very likely contribute to this phenomenon. It is important to ask why the RyR2 channel has to be phosphorylated, when this post-translational modification is not a prerequisite for RyR1 and RyR3 inhibition by dantrolene. This could be explained from the standpoint of structural biology. A detailed analysis of conformation changes in the cryo-EM 3D structure of all three RyR isoforms caused by phosphorylation and/or the binding of FKBP12/12.6, CaM, ATP analog, and cytosolic Mg^2+^ will provide crucial insight into allosteric interactions likely regulating conformation and accessibility of the dantrolene-binding site. The answer to the question surrounding dantrolene selectivity will revolutionize the future of dantrolene in clinical pharmacotherapy of cardio- and neuropathologies as RyR2 hyperphosphorylation has been implicated under various pathological conditions. Because dantrolene is emerging as a novel promising therapeutic agent, there is a pressing need for new cost-effective dantrolene derivates and formulations with superior water-solubility, which will mainly improve oral bioavailability. In addition, dantrolene therapy should be organ-specific; therefore, dantrolene delivery has to be optimized to reduce serious side-effects.

## Figures and Tables

**Table 1:** Effect of dantrolene on the RyR1 channel in cell-free experiments. ATP, Mg^2+^, CaM, and dantrolene were added to the RyR1 cytosolic face.

Effect of Dantrolene		Presence		Temperature	RyR1 Type	References
	ATP or Analog	Mg^2+^	CaM			
Yes	▪	▪	ND	RT	MH	[87]
No	▫	▫	ND	RT	MH	[97]
No	▫	▫	ND	37 °C		
No	▫	▫	ND	RT	WT	[28]
Yes	▪	▪	ND	RT		
No	▪	▫	ND	RT	WT	[120]
No	▫	▫	ND	RT	WT	[89]
No	▪	▪	ND	RT		
No	▪	▫	▪	RT	WT	[118]
No	▪	▫	▪	35 °C		
No	▫	▫	ND	RT	WT	[121]
Yes	▫	▫	▪	RT		
No	▪	▫	▫	RT	WT	[122]
No	▪	▫	▪	RT		
Yes	▪	▪	▫	RT		
No	▪	▫	ND	RT	WT	[119]
Yes	▪	▫	ND	37 °C		
Yes	▪	▫	▪	RT		
No	▪	▪	ND	36 °C	MH, WT	[29]
No	▪	▪	▪	19 °C	MH	
Yes	▪	▪	▪	36 °C	MH, WT	
No	▫	▫	▪	37 °C	MH	[30]
Yes	▪	▫	▪	37 °C	MH, WT	
Yes	▪	▪	▪	37 °C	MH	

ND, not determined; RT, room temperature; WT, wild-type; ▫, absent; ▪, present.

**Table 2:** The values of dissociation constant for dantrolene binding to the RyR isoforms.

RyR Isoform	K_d_ (nM)	Experimental Approach	References
RyR1	365 ± 50	[^3^H]dantrolene binding	[124]
	277 ± 25	[^3^H]dantrolene binding	[18]
	5	[^14^C]dantrolene binding	[125]
RyR2	2000	[^14^C]dantrolene binding	[125]
RyR3	~K_d_ of RyR1	Inhibition of [^3^H]ryanodine binding	[30]

K_d_, dissociation constant.

**Table 3:** Effect of dantrolene on the RyR2 channel in cell-free experiments. ATP, Mg^2+^, CaM, and dantrolene were added to the RyR2 cytosolic face.

Effect of Dantrolene		Presence		Temperature	RyR2 Type	References
	ATP or Analog	Mg^2+^	CaM			
No	▪	▪	▫	36 °C	WT	[29]
No	▪	▪	▪	36 °C		
No	▪	▫	▪	37 °C	WT	[30]
No	▫	▫	▫	RT	WT	[121]
Yes	▫	▫	▪	RT		
Yes	▫	▫	▪	RT	WT	[92]
No	▪	▪	ND	35 °C	WT	[31]
Yes	▪	▪	ND	35 °C	WT-P	
Yes	▪	▪	ND	35 °C	WT-FH	[31]

ND, not determined; RT, room temperature; WT, wild-type; WT-P, phosphorylated WT, WT-FH, WT isolated from failing hearts; ▫, absent; ▪, present.

**Table 4:** Effect of dantrolene on the RyR3 channel in cell-free experiments. ATP, Mg^2+^, CaM, and dantrolene were added to the RyR3 cytosolic face.

Effect of Dantrolene		Presence		Temperature	RyR3 Type	References
	ATP or Analog	Mg^2+^	CaM			
No	▪	▫	▪	RT	WT	[30]
No	▫	▫	▪	37 °C		
Yes	▪	▫	▪	37 °C		

RT, room temperature; WT, wild-type; ▫, absent; ▪, present.

## Data Availability

Not applicable.
